# Challenges and adaptations to public involvement with marginalised groups during the COVID-19 pandemic: commentary with illustrative case studies in the context of patient safety research

**DOI:** 10.1186/s40900-022-00345-x

**Published:** 2022-04-11

**Authors:** Isabel Adeyemi, Caroline Sanders, Bie Nio Ong, Kelly Howells, Leah Quinlivan, Louise Gorman, Sally Giles, Mat Amp, Elizabeth Monaghan, Sumaira Naseem, Adam Pearson, Sudeh Cheraghi-Sohi

**Affiliations:** 1 NIHR Greater Manchester Patient Safety Translational Research Centre, Manchester Academic Health Science Centre, The University of Manchester, Manchester, UK; 2 NIHR School for Primary Care Research, The University of Manchester, Williamson Building, Oxford Road, Manchester, M13 9PL UK; 3 NIHR Applied Research Collaboration, Greater Manchester, UK; 4 Centre for Mental Health and Safety, University of Manchester, Manchester, UK; 5 Groundswell, London, UK

**Keywords:** Patient and public involvement, Reflections, Marginalised groups, Patient safety

## Abstract

Patient and public involvement (PPI) is integral to research on patient safety in the NIHR Greater Manchester Patient Safety Translational Research Centre (NIHR GMPSTRC), and is central to our patient safety research within our theme focusing on people in marginalised groups. Due to the impact of COVID-19, researchers had to adapt how they *do* PPI. For marginalised groups, remote working and digital adaptations (the key adaptations made in accessing and utilising health services in the United Kingdom during COVID-19) can potentially lead to further marginalisation of people already marginalised and provide new barriers to others. This editorial showcases three case examples of PPI with marginalised groups during COVID-19, these are with: (1) adults with vision impairments, (2) adults and carers with lived experience of self-harm and/ or suicide and (3) adults with lived experience of homelessness. In these case examples, we focus on challenges relating to key aspects of PPI during the pandemic. First, setting up a PPI advisory group and secondly maintaining relationships and effective PPI with a pre-existing advisory group. We contrast these examples using more traditional ways of ‘doing PPI’ i.e. involving public contributors in various stages of the research cycle, with a more fully ‘co-produced’ approach to research when developing a new patient safety intervention. Important considerations for PPI with marginalised groups during COVID-19 include: how to avoid exacerbating the digital divide when using video conferencing for PPI, the need for enhanced awareness around flexibility and resources, and the value of working closely with specialist charities to enable adaptations that are sensitive to the changed circumstances and needs of PPI contributors.

## Plain English summary

The National Institute of Health Research (NIHR) Greater Manchester Patient Safety Translational research Centre (GMPSTRC) carries out research to improve patient safety. We work in partnership with patients and members of the public to plan, manage, design, and carry out the research. This is labelled as patient and public involvement. A key area of GMPSTRC research focuses on people who may be marginalised from healthcare and potentially suffer increased patient safety risks relative to the general population. COVID-19 impacted on research in multiple ways, notably *how* researchers work with members of the public. Remote working via the use of internet enabled technology has now become more commonplace, although this raises concerns about digital exclusion and how to address barriers when face-to-face PPI is not possible. This article showcases three examples of how researchers and public contributors with lived experience have adapted to working together during the COVID-19 pandemic. These case examples are: (1) setting up a public involvement group for developing an intervention to reduce medication errors for adults with vision impairments; (2) working with a pre-existing group of public contributors who are adults and carers with lived experience of self-harm and/or suicide; and (3) working with a charity called Groundswell who train and support researchers with lived experience of homelessness. Challenges during COVID-19 have been making video conferencing meetings accessible to members who have a range of different needs, and addressing the digital divide by allowing members flexibility in how they contribute to research. The article provides examples of how these challenges were addressed.

## Background

Marginalised communities, may be defined as “individuals, groups or populations outside of ‘mainstream society’” [1]. According to Beresford [2], individuals and communities meeting this definition, may be excluded on the basis of:

- equality issues in relation to gender, sexuality, race, class, culture, belief, age, impairment and more;
- where they live, for example, if homeless or in prison;
- how they communicate, for example, if English is not their first language;
- the nature of impairments, in terms of when these are seen as too complex or severe for the person to contribute;
- and/or where they are seen as ‘unwanted’ voices in that they are considered disruptive to mainstream discourse.

Marginalised groups are least likely to be represented in research studies [3] and are often described as “hard-to-reach” or seldom heard. This means that the views, priorities, and preferences of patients, carers, and the communities that support them are not systematically used to improve the care they themselves receive. The COVID-19 pandemic has disproportionally affected certain demographics, for example people from ethnic minority groups or those from socially disadvantaged or deprived communities face greater health risks and poorer health outcomes due to direct or indirect COVID-19 effects [4]. The impact of the pandemic on marginalised groups risks exacerbating existing health and social inequalities, or creating new ones [5]. Taken together, these factors make doing research on, and with, marginalised groups more important than ever.

Nationally, the promotion of a diverse and inclusive public involvement community is at the core of the United Kingdom’s government’s vision for a patient-centred National Health Service [6] and the National Institute for Health Research’s (NIHR) vision of public involvement for 2025 [7]. Internationally, enabling co-production and community participation with marginalised people has been highlighted as especially important in the global health context of COVID-19 [8, 9]. However, at the start of the pandemic, research and associated PPI activities had either stopped or were happening late(r) in the research development process when opportunities for patients and the public to contribute meaningfully to research design are reduced [10]. The drop in these activities is thought to be partly due to researchers’ assumptions about public involvement in a pandemic, for example: that public contributors would not be as motivated or available, or that public involvement groups would not be operating because their usual work had been drastically disrupted [10]. Researchers also had to respond at short notice to calls for rapid research before adaptations were in place to facilitate involvement.

A range of approaches involving or engaging with the public exist and our work prior to and during the COVID-19 pandemic, has drawn on these. ‘Traditional’ methods of PPI often comprise some form of face-to-face interaction, either at an individual level or at a group level [11]. Such interactions can include regular meetings e.g. project advisory groups, reference groups, public events or workshops [12]. Another example is the community based participatory research, an approach that optimizes community engagement through partnerships between community members, organizational representatives, and researchers [13, 14]. Both traditional and community based participatory research approaches entail relationship building between academics, community members, and organizations in order to develop involvement throughout the research cycle. When implementing PPI, Blackwell and Rowbottom [15] highlight three challenges. The first challenge is the difficulty in reaching people with first-hand experience. The second is avoiding distress to PPI contributors in that participating in PPI events can bring up difficult memories and strong emotions for some people. The third challenge is exacerbating further marginalisation by excluding participation due to use of technology, location of meetings or lack of funding for expenses. These challenges might be intensified during the pandemic and we present case studies from our patient safety research to illustrate them and to explore learning from adaptations made to enable PPI during the pandemic. We discuss our experiences within the broader literature to draw lessons for future PPI in research.

## Case studies for PPI with marginalised groups during COVID-19

The research focus of the NIHR GMPSTRC is on developing and testing new approaches to patient safety in primary care and across transitional care settings in the community, social care, and hospitals. Populations of marginalised groups working with the NIHR GMPSTRC were identified from our earlier literature review work [16]. The three case examples were selected to highlight key aspects of PPI steps and/or processes:

1. Commencing PPI: setting up a public involvement group on a research study,
2. Maintaining and adapting PPI: new ways of working with a pre-existing group of public contributors,
3. Partnership working: adapting and involving research in partnership with an established charitable body.

## Case study 1: setting up a PPI group and conducting an online event during the COVID-19 pandemic with adults with vision impairment

### Context

Our research aims to co-design a digital/online resource with people who are vision impaired and with pharmacists [17]. Vision impairment (VI) is a term used to describe any kind of vision loss, whether it is someone who cannot see at all or someone who has partial vision loss. The online resource is for pharmacists to help people with VI to manage their medicines safely. Medication safety is a key area of patient safety and people with vision impairments have more challenges in safely managing their own medications versus the sighted population [18]. During the COVID-19 pandemic, we set up a PPI advisory group comprising of adults with vision impairments and a meeting with adults with vision impairments and pharmacists to co-design a training resource for pharmacists.

### The process for setting up the PPI group

We sought specialist input for this project for two reasons: (1) VI was a new area of research for the majority of the research team and (2) VI encompasses a range of needs and challenges that are well understood by the Royal National Institute of Blind People (RNIB) as this is a national charity offering information, support and advice to almost two million people in the UK with sight loss. The RNIB provided expert input.

Our initial engagement revealed multiple opportunities for researchers to ensure that their approaches to PPI could be inclusive to people with VI. Key aspect were knowledge sharing on VI itself and the best ways of involving people via remote methods. Two representatives from the RNIB provided a 1-h training session to researchers on what is it like to have a vision impairment and the day-to-day barriers people with vision impairments may face. The training sessions raised awareness on the language used to describe vision impairments and highlighted the myths around these conditions. The training enabled us as researchers to identify our own misconceptions about vision impairments and to think about what support PPI contributors may need to fully participate in any PPI activities. Importantly, in terms of doing PPI during the pandemic, we needed to know *how* we were going to be able to interact successfully with people with VI remotely. It provided guidance on how to facilitate a digital group discussion with people who have vision impairments using video-conferencing. Microsoft Teams was considered the preferred choice of online meeting platform due to features found to be useful to adults with vision impairments. RNIB provides Microsoft Teams training to their members and therefore users would be familiar with the system. Whilst we may have come to these learnings ourselves, learning from the RNIB’s adaptations of their methods of working during the pandemic meant that we were able to *do* PPI in the most efficient and appropriate manner.

Once we had learned about the intricacies of remote involvement with adults with VI, we needed to then be able to communicate our research study and involvement opportunities. Recruiting via the usual channels e.g. attending face-to-face groups and explaining research however was not an option. The RNIB agreed to promote the PPI opportunities through their channels. They have a Northwest Community Connection Team that enabled people with VI to access the PPI opportunity. This meant that our research could be established through a trusted and well recognised source. The reach of the RNIB far exceeded what we might have been able to do locally. Five PPI contributors with vision impairments were recruited in October 2020. All but one of the PPI contributors did not have any experience of public involvement in research. Degrees of sight loss among PPI contributors varied with some having limited vision, and others having no vision. At the first PPI meeting, we discussed PPI contributors’ preferences for meeting frequency and communication methods in-between group meetings. Whilst this is the case in setting up PPI groups generally, people with VI had additional needs. For example, whether they preferred that documents be sent in large written text or in an audio file. These issues were revisited regularly in case amendments were needed as the group progressed.

### The process for setting up the co-design event

The PPI advisory group met on four occasions prior to the co-design event. Setting up the event with adults with vision impairments and pharmacists required more time and resources compared with a face-to-face meeting. This was largely due to the technical challenges in identifying and accommodating the digital needs of the contributors. RNIB provided further training to co-facilitators on the running of the co-design meeting. Co-facilitators also met with the lead researcher on two occasions to ensure everyone felt comfortable with their role and understood how to use Microsoft Teams.

Eight adults with VI and six pharmacists were invited to the two-hour co-design meeting. Break out rooms comprised of two pharmacists and two adults with vision impairments each. Each break out room comprised of an adult with severe sight loss, and another adult with vision impairment/s who had some level of sight remaining. The break out rooms needed to contain fewer people to make it more accessible to adults with vision impairments and to ensure enough time for each person to speak. Through turn taking the co-facilitator invited attendees to speak thus avoiding reliance on visual cues. Attendees were asked to mute themselves on Microsoft teams when it was not their turn to speak. Each break out room had two co-facilitators: one led the session and invited attendees to speak, the other took notes to summarise the points at the end of the session. Electronic post-its and white board software were not used because this was deemed inappropriate for adults with vision impairments. Instead, frequent spoken summaries and paraphrasing was used to remind attendees of the points talked about.

### Reflections on setting up the PPI advisory group and the co-design event

Individuals with different VI have to use different techniques and adaptations to enable them in their everyday life. Compared with our experiences of running PPI groups in population groups *without* VI, examples of our adaptations for working remotely with people with vision impairments are outlined in Table 1:

**Table 1:** Adaptations of PPI activities with adults with VI during the COVID-19 pandemic

Activity	Adaptations
Planning PPI activities	Link with a specialist organisation which has experience of working with the target population and who can provide training to researchers for planning PPI activities Choose the appropriate technology/adaptations and level of support: e.g. The decision to use Microsoft Teams and not Zoom, number of facilitators, training facilitators on needs of population and technology Use the most appropriate visual and audio set up e.g. yellow background and for the speaker to wear dark clothing. This is because yellow is one of the last colours individuals are able to see when losing their sight and the dark clothing provides a contrast to allow the speaker to be seen more clearly
During PPI meetings	*Adapting the communication formats:* Doing verbal introductions when all contributors are present or repeating introductions when a contributor joins the meeting in order for everyone to know who is in the meeting Stating your name *every time* upon speaking; not using the chat function in Microsoft Teams because this can be confusing when also using screen readers If you ask a question then address the person/people you are asking it to by name, invite that person to speak. When listening to a person with vision impairments speak, use audio cues rather than non-verbal cues such as nodding your head in agreement Any images used in the presentation need to be audio described appropriately
After PPI meetings	The format of how information is sent needs to be flexible. We asked adults with VI about their preferred format for how they would like to receive information, for example in large format via email, audio format, in braille. This can be resource and time intensive but is essential to meeting individual needs for inclusivity

Two PPI contributors reflected on their experiences of being part of the remote PPI group. The contributors had different views on the accessibility of virtual meetings. In comparing virtual and face-to-face meetings, there was no clear preference. Differences in preference for virtual versus face-to-face meetings are because people with VI have distinct ways of processing information and may be situated differently on the spectrum of sight loss. The biggest contrast is between those with useful vision and those with no useful vision (totally blind). The perceived benefits of virtual meetings in comparison to face-to-face meetings are: people with VI may have other health conditions and may not be able to attend physical meetings; they are useful for people with VI who are proficient in using digital formats and prefer electronic material; they enable people with VI who live far away to take part in research development. Ultimately, the differences in perspective depend on individual impairments and people’s contexts.

For some people with VI, communication can be easier in person. One PPI contributor elaborated that if you were to close your eyes you can still feel who is around you, and this facilitates communication. The challenges of virtual meetings are:

- that some people with VI may make use of tactile information, and this information cannot be translated into another sensory input such as audio. Moreover, attendees cannot receive alternative information on visual cues, such as haptic feedback.
- That people with VI require different levels of description for items/ images. This is easier to provide in person because a physical meeting has reference points that can be used metaphorically. For example, a person who is deaf/blind relies heavily on tactile information for communication, and without this, virtual meetings are limited.
- The lack of accessible specialist software and training for people with VI is a barrier for conducting virtual meetings. For example, online platforms do not usually include built-in functions such as screen reader text that translates text and image content into speech or braille. A further barrier is the financial cost for people with VI. In summary, it takes additional time and money for a vision impaired person to access a virtual meeting at the same level as a non-vision impaired person.

### Outcomes

The pandemic created uncertainty in terms of the viability of setting up a PPI group and co-design event, for people with VI. It proved possible and despite difficulties researchers and the PPI contributors achieved good levels of involvement. Group coherence was made easier through continued expert support from the RNIB and that some PPI contributors knew each other already through RNIB. This was the first project they had worked together on and the first using virtual methods. However, both researchers and PPI contributors feel that virtual meetings are less able to respond to some individual needs related to different VI. We have learned through carrying out the meetings virtually that remote meetings are possible with this population group and should be offered alongside face-to-face meetings where feasible.

## Case study 2: maintaining an established PPI group of adults and carers with lived experience of self-harm and/or suicide

### Context

The challenge of minimising distress from PPI activities is not new in the context of PPI with marginalised groups. Studies carried out prior to the start of the pandemic have shown a higher prevalence of psychiatric disorders in marginalised groups compared with the age-matched general population [19, 20]. The PPI group in the mental health theme of the NIHR GMPSTRC and in the Centre for Mental Health and Safety contributes to a broad range of research studies. We established the PPI group “Mutual Support for Mental Health Research” (MS4MH-R) in November 2018. PPI contributors have lived experience of self-harm, suicidal ideation, and serious mental distress. We had established a robust and flexible system for PPI prior to the pandemic that was working well for all contributors. There were regular face-to-face meetings- around four meetings per year for the whole group- and project specific meetings took place more frequently. At the outset of planning our PPI strategy, we provided several modes for involvement because some people may become unwell, experience social anxiety, or have mobility issues. These included email correspondence, social media correspondence, and telephone contact. Prior to the COVID-19 pandemic, video conferencing meetings were not used as a means of remote PPI so this was a new experience for all involved.

Self-harm and suicide prevention are delicate research areas, leading to concern that discussing sensitive topics may cause distress or lead to further self-harm/ suicidal behaviour [21]. Whilst participation in self-harm/suicide prevention research may benefit some individuals [21, 22], care is needed because some participants find discussing health service experiences can trigger negative emotions and low mood [23]. Thus, well-being is an essential foundation for PPI in this area. Prior to COVID-19, a series of individual-level well-being plans were co-designed for each PPI contributor as a way of managing their personal distress, should any arise. The well-being plans focussed on what to do if PPI contributors become unwell and who to contact.

### PPI during the COVID-19 pandemic

During the COVID-19 pandemic, the use of video conferencing became our main and new means of facilitating involvement activity. Remote meetings/presentations were designed taking into account well-being, PPI activity and digital access issues (e.g., some people may need/prefer to join meetings by phone).

We provide bi-monthly written updates and frequently check with each PPI contributor about their communication preferences. Within the video-conferencing meeting, we ensure there is a flexible agenda allowing time for well-being updates, building rapport, and frequent comfort breaks. In this way, the video conferencing meetings mirrored face-to-face meetings. Each meeting has two facilitators who are researchers. One chairs the meeting, welcomes PPI contributors, informs them of the content of the meeting in case there are any potentially distressing/triggering topics, and reaffirms the agenda and expectations for the meeting. The second researcher focuses on well-being in the meeting and within the chat function.

During the COVID-19 pandemic, the well-being plans and terms of reference were revisited. No major changes were needed to the plans except *how* well-being was monitored and adapted for remote working. For example, if a PPI contributor turned off their cameras or logged out of the meeting abruptly, this acted as a prompt for the researcher to check on the contributor in case this action was triggered by the content of the discussion. Some PPI contributors wanted their video off because of IT issues, sensory issues, migraines, or privacy. Video conferencing meetings require the management of multiple factors such as the technology, picking up non-verbal cues, turn-taking difficulties, using written ‘chat’ instead of speaking. All of these factors may be amplified for people who experience anxieties. However, PPI contributors felt that some of these issues were mitigated by the established relationships between researchers and contributors. Similar to face-to-face meetings, we operated an open door policy for online meetings where PPI contributors can leave during sections of the meeting and they can return when they want to. The researcher manages admittance to and departure from the meeting. Finally, following the meeting, both researchers contact PPI contributors to check well-being within 24 h and to ensure that payment forms are completed. Feedback is encouraged after each meeting and any suggested changes implemented where possible. Table 2 summarises the key adaptions.

**Table 2:** Adaptations of PPI activities with adults and carers with lived experience of self-harm and/ or suicide during the COVID-19 pandemic

Activity	Adaptations
Before PPI meetings	Consider adapting well-being plans when designing remote meetings and presentations Give PPI contributors different involvement options (e.g. via video conferencing or through the chat mode, via telephone)
During PPI meetings	Have an “open door” policy where PPI contributors can exit sections of the meeting that they may find distressing but can return for the next section of the meeting Have two facilitators- one who chairs the meeting, another who checks in with PPI contributors via the chat function Check in with PPI contributors who turn their camera off or exit the meeting
After PPI meetings	Do well-being checks with PPI contributors within 24 h of meetings

The needs of PPI contributors and researchers in video conferencing meetings were different to face-to-face meetings (e.g., concentration levels, zoom fatigue, communicating virtually), but we all rapidly learnt and adapted. Despite some challenges (e.g., lack of face-to-face contact, communication difficulties), video conferencing did provide some new opportunities. It enabled some PPI contributors with mobility issues to engage more easily in involvement activities. For example, one PPI contributor with hearing loss could connect her hearing aid to the computer and this reduced the impact of background noises and made it easier for her to engage with the meetings. Attending remotely might reduce anxiety for some PPI contributors because they could attend the meeting from home that is a safe space. Reducing the need to travel can stimulate engagement and through remote access, an additional eight PPI contributors from a wider geographical area were recruited during the pandemic. They had found information about the PPI group online and contacted the research team.

Inclusivity and accessibility of our PPI work is a topic we continuously reflect upon [24]. Accessibility to involvement was considered pre-pandemic, but with the advent of the pandemic an audit of digital-divide issues was considered important. The group needed to be able to access freely available and user-friendly software. One of the key issues concerned digital connection (such as internet availability and technology to access the internet). To ensure that those without the necessary technology could effectively contribute, researchers facilitated PPI contributors’ involvement in different ways. For example, in qualitative coding workshops, PPI contributors needed to have access to Zoom, Microsoft word, and Open-Source software. PPI contributors also needed to become familiar with the software to code the data. A solution to the digital divide was to allow flexibility in *how* PPI contributors returned their coding (including paper and pen coding returned via post). The different adaptations for individual needs were time consuming and onerous for the researchers. The flexible approach to meeting participation, and the additional engagement work by phone and email in between video-conferencing meetings were effective in tackling the digital divide.

Finally, two key challenges during the pandemic were: first, new bureaucracy for ensuring that payment for involvement was processed easily and quickly meant setting up a new process for accepting electronic signatures. Electronic payment forms had pre-filled sections that made completion easier. Second, enabling group cohesion when new contributors join an existing group was more difficult and required heightened sensitivity by the researchers.

## Case study 3: an example of a participatory approach to co-producing a Covid-19 research study to explore experiences of changes to support access to primary care for people experiencing homelessness during the pandemic

### Context

Studies indicate that people experiencing homelessness have higher levels of co-morbidity and shorter life expectancy than the general population, but are less likely to have access to safe and quality healthcare [25]. Poor access to primary care is a major concern, sometimes due to organisational and bureaucratic barriers, but also because of stigma [26]. Additionally, lack of basic amenities to support treatments such as being able to store medication, or to prevent and manage infections compound problems. Against this back-drop, questions need addressing regarding whether recent changes to service delivery in the context of COVID-19 affect accessing care and inequalities in health outcomes.

This project adopted a participatory and action research approach that has been widely used for conducting community based research, including amongst homeless people and service providers [27–29]. Action research and participatory methods refer to styles of research that emphasise collaboration and democratic working between multiple partners to bring about change [30, 31]. We partnered with a national homeless charity, Groundswell, to implement a participatory ‘peer led’ research model, in which interviews are carried out by a researcher with lived experience of homelessness. Groundswell also provide emotional and practical support to their researchers as peer researchers may have to relive their personal experiences and therefore opportunities to debrief and discuss their mental wellbeing are essential. In addition to training, supporting, and employing peer researchers, Groundswell also have an established advisory group for research, comprising of ten people with lived experience of homelessness.

### PPI during the COVID-19 pandemic

We needed to understand how COVID-19 was impacting on access to care for homeless people and how the research study may need to be adapted. We consulted the Groundswell advisory group, GP practices, and homeless and vulnerable adults’ services. Together with the Groundswell researchers, we amended the scope of the research study within the context of the pandemic. Prior to the pandemic, there was resistance from stakeholders in developing digital interventions for homeless adults because they thought these could increase health inequalities. Although face-to-face contact between certain groups of healthcare professionals, hostel workers, and homeless adults did not stop during the pandemic, remote consultations in general practice were mandated. This change suggested that the research would need to explore remote and digital interventions in this population group.

Working together pre-pandemic meant that we already had a successful team and were following guiding principles for co-production, including ‘sharing power’, reciprocity, respecting and valuing knowledge within relationships [32]. The strong team relationships continued during the Covid-19 pandemic and enabled us to address new challenges of involving the Groundswell public advisory group, and recruitment of participants. The trusted relationships developed with the advisory group prior to the pandemic made adaptations to remote working easier. All contributors of the group had ability to attend meetings online with introduction and support for using the online platform provided by Groundswell. However, their lived experience of homelessness meant they could readily advise on the research, including digital barriers.

A key impact of the pandemic was on the Groundswell researcher’s role and their ways of working. Before the pandemic, the Groundswell researcher visited daycentres to discuss the research, read through the participant information sheet and established rapport with participants. During the pandemic, the Groundswell researcher linked with key staff who were continuing face-to-face services, including community nurses and hostel workers. These staff acted as a trusted link to introduce the research by distributing the co-designed ‘easy access’ participant information sheets.

Some people did contact the researcher to offer participation but, had not read through the information sheet. When the Groundswell researcher read through the information sheet over the phone, people with lived experience of homelessness were sometimes sceptical and hung up. For example, one participant indicated he was worried the research was a ‘scam’. In adapting their ways of working during COVID-19, the Groundswell researcher reflected that lived experience is particularly important in communication over the phone. With face-to-face contact, barriers are easier to break down through eye contact and rapport. In contrast, the Groundswell researcher felt that on the phone participants can easily picture someone who is removed from their world. To counter this, the Groundswell researcher discussed his lived experience with participants earlier on in the phone conversation. It was also important to be mindful of potential anger from participants as COVID-19 was highlighted as yet another pressing crisis joining multiple crises that make up their days.

The Groundswell researcher was worried the remote approach could result in higher rates of disengagement because of the length of the consent process combined with lack of trust and clarity surrounding the identity of the researcher. However, the service providers collaborating with the team volunteered to read through the information sheet with participants rather than just give them out. These staff also helped to arrange a suitable time for telephone interviews and this gave the Groundswell researcher more time for an informal conversation with participants prior to the interview. Importantly, there was maximum flexibility for timing of calls, and these were conducted by telephone and did not depend on ability to connect to and use an internet platform.

The involvement of service providers was also fundamental in helping Groundswell pay participants for their involvement in the research. Prior to COVID-19, people participating in the research were generally reimbursed in cash. However, during the pandemic, supermarket vouchers were instead posted to participants. Most participants lived in hostels and not many participants involved in the research were street homeless (e.g. with limited contact with a hostel). Vouchers were posted directly to the service providers or were emailed to participants where appropriate.

Following data collection, the impact of COVID-19 on the research study was minimal. All interviews were co-analysed by the University and Groundswell team using Framework analysis. Documents were shared via Dropbox for business. Regular meetings were held via zoom to discuss findings with the wider research team. This led to the co-authorship of a recommendations document relating to remote consultations for people experiencing homelessness. We were able to hold an online public involvement event in partnership with Groundswell and co-facilitated by an additional Groundswell researcher. PPI contributors who were part of Groundswell’s advisory group helped to further develop and refine a guidance document for use in primary care based on our research findings.

This case study addresses avoiding further marginalisation by excluding participation due to either use of technology or location of meetings. This case study illustrates working together with people with lived experiences of homelessness and key stakeholders through all stages of the project by adapting study design, data collection, data analysis, authorship of outputs. In addition to this case study, the Groundswell researcher and co-author, has published more in-depth reflections on working on this research study [33]. The case study highlights the importance of university researchers and Groundswell researchers working together within the context of the pandemic by making changes to co-production, and building trust in the working relationship. Table 3 summarises the key adaptations for case 3.

**Table 3:** Adaptations of a participatory approach with adults with lived experience of homelessness during the COVID-19 pandemic

Challenge	Adaptations
Ensuring that the research remains relevant with regards to changes in the research environment e.g. primary care during the pandemic	The scope of the research was amended due to changes in primary care during the pandemic. This ensured that the research remained relevant to people with lived experience of homelessness and to stakeholders
Researcher difficulties in building rapport with participants when they could not meet face to face	The Groundswell researcher shared information about themselves and their lived experience of homelessness early on in the conversation when speaking to participants over the telephone
Digital barriers to recruitment of research participants and involvement of public contributors with lived experience	Gatekeepers such as hostel workers distributed the participant information sheet, introduced the study, and arranged a time for the Groundswell researcher to call Telephone interviews were conducted with maximum flexibility and did not depend on ability to connect to and use an internet platform For reimbursement, vouchers were posted to staff (e.g. hostel workers) or were emailed directly to participants where appropriate The Groundswell advisory group were supported to meet online

## Discussion

Our work with marginalised groups during the pandemic illustrates that doing PPI remotely is possible, acceptable and can achieve successful outcomes. Remote methods provided new opportunities for involvement for some and new ways of working which were seen as advantageous in certain contexts and/or for certain people. However, there are also some ongoing challenges and limitations.

The first and largest limitation surrounds the issue of inclusivity due to the issue of digital poverty. The COVID-19 pandemic has more explicitly exposed digital poverty with poor access to digital devices and inability to afford data plans and internet connection [34]. Such barriers have been particularly apparent for marginalised groups requiring consideration for enabling remote PPI during COVID-19. The digital divide means that in relying on virtual resources, certain groups such as rural groups, ethnic minorities, migrant groups or those from poorer socio-demographic backgrounds may be excluded due to an inability to access or effectively use digital platforms [35–37]. Case 1 for example, highlights how technology may reduce the inclusivity of involvement opportunities for some people with VI vs face-to-face formats, however this was minimised to a large degree by the support provided by the RNIB. Such support aided researchers’ understanding of the needs of this population, advising on the most appropriate technology and how best to use it. Case 2 also illustrated examples of digital poverty issues and how this was managed via the various strategies such as freely available software and non-video conferencing options for involvement opportunities. Additionally, our work on homelessness serves as an example of steps taken to maximise inclusion of participants where digital poverty is a common aspect of experience. In this setting, people may have access to mobile phones but they may be basic phones with limited functioning, or they may be unable to purchase data or have easy access to charge devices. We were able to maximise inclusion of people with varied degrees of digital engagement by working closely with service providers to enable face-to-face recruitment, and by maximising flexibility for telephone interviews that were not dependent on internet connection or high specification of device. Working in partnership with Groundswell, also enabled us to work with their long established advisory group for specialised public involvement, and to share learning from their wider work to engage with people experiencing homelessness who are often digitally excluded (e.g. their work to capture stories and everyday experiences of COVID-19 through ‘on our radar’ project [38]). Whilst this example is specific to the context of homelessness, the approaches to addressing these challenges are relevant to wider populations and communities where digital poverty is apparent. However, it must be acknowledged that digital approaches can and will exclude those unable or unwilling to engage with them and that this remains an issue for involvement more widely, *how* to engage the most marginalised, whatever the method(s).

Secondly, there is the issue of developing trust and authenticity of involvement remotely. All cases highlighted the importance of this and had different approaches to its management. Case 2 illustrated how trust and authenticity established over-time and pre-pandemic, were critical to the mitigation of any anxieties associated with remote involvement. Case 1 illustrates how creating a new PPI group and doing significant involvement activities remotely was possible but that key to this was the partnership working with the RNIB. Partnering early and with an established national charity, aided both the authenticity of the work and the trust of potential contributors when for example the PPI opportunities were promoted through their official channels. It was difficult to comment however on whether relationships within the PPI group were felt to be as authentic as face-to-face meetings due to the newness of PPI for group contributors. Case 3 also highlighted that community based approaches to PPI emphasise that in-person interactions are central to building trusting relationships [39]. They responded to these challenges of remote working by highlighting that flexibility is already embedded in a community based participatory research approach [40]. Our work with adults with experience of homelessness shows the impact remote working had on the Groundswell researcher building rapport with participants they were recruiting and what adaptations had to be made. Going forward, face-to-face meetings are still preferable to remote methods for this group in terms of building trust and rapport. From these case examples, it is clear that strong partnership working with relevant charities and/or specialist organisations we were able to minimise the first and third challenges highlighted by Blackwell and Rowbottom [15].

Finally, and practically, social distancing restrictions made it necessary to stop providing payments in cash, and to consider virtual methods of PPI payments to marginalised groups [35]. In some cases, however, virtual methods of payments such as credit and vouchers may not be accessible for those who are digitally excluded. In the case of adults with lived experience of homelessness, this barrier was addressed by working in partnership with Groundswell’s peer researchers, and with service providers who were gatekeepers to accessing homeless adults during the pandemic.

There are however also advantages to working remotely with PPI contributors. Case 2 illustrates how remote methods can offer new and more flexible opportunities for involvement, which for some people can reduce the issues and anxieties associated with face-to-face involvement (e.g. travelling to meetings). Case 2 also provides an example of how any distress associated with PPI activities may not be avoidable (as with face-to-face methods) but that well-being can be monitored differently, with technology providing new triggers for real-time well-being checks. Such advantages are also noted in the literature with respect to the ability to engage populations/individuals that may not always have the time or capacity to be involved in research [5]. Basic strategies to reduce the barriers to involvement in PPI meetings and workshops have included organising PPI workshops at different times of the day, and offering assistance and practice in Zoom calls for anyone new to using Zoom [41]. Archibald and colleagues [42] argue that the increased use of digital technologies for research is a result of technical advances, opportunities for wider inclusion of participants, and can act as complement to traditional methods. In their study of employing Zoom for research interviews, they found that establishing rapport, convenience (saving time, distance not being a problem), and simplicity of the technology were key advantages. While they encountered some technical difficulties, both researchers and participants concluded that Zoom widened participation and offered flexibility. This type of study highlights the trend of using digital methods more widely and helps to normalise remote PPI research activities.

In summary, whilst we have gained certain insights from doing PPI differently as a result of COVID-19, we feel that there are overarching key recommendations from our experiences of that are applicable more generally and these are:

- Being maximally flexible. Researchers must be prepared to accommodate the specific needs of individuals in terms of the approach to remote involvement. Having a range of involvement approaches is key to inclusivity and mitigating issues around digital poverty and accessibility of PPI opportunities.
- Remote involvement can be successful but harder than face-to-face involvement for certain people and for certain contexts, with trust and relationship building being more difficult. This can be mitigated by partnering with established and trusted people and/or organisations.
- Link early on with charities and specialist organisations who already work with the target population to plan activities, how remote involvement might work for the group and what technologies might or might not be appropriate.
- Research costs, and funders, should consider the extra time and resources that providing flexible involvement options require to allow for increased involvement opportunities. This includes both researcher time and the time of those involved in PPI. In particular, and beyond the reimbursement of individual PPI contributor time, is the time and effort of the charities and/or organisations researchers work with in order to conduct their PPI activities. Such valuable efforts by such organisations, should i) be recognised in itself in terms of the impact in enabling relevant and successful PPI (and research more widely) and ii) also directly in monetary terms to support marginalised individuals not only to participate in PPI opportunities but as financial support for wider organisational operations in an increasingly pressurised socio-economic environment.

## Conclusion

It is vital that researchers and public involvement practitioners review and share best practice to ensure that public involvement throughout and beyond the pandemic is optimised and that any learnings can be addressed for future developments. Our article provides three case examples of PPI and co-production with marginalised groups. The biggest adaptations to PPI activities were using video-conferencing meetings whilst not exacerbating the digital divide. To counteract this danger, partnerships with specialist charities enabled adjustments that were sensitive to the changed circumstances and needs of PPI contributors. Post pandemic, we recommend that video-conferencing meetings should remain an additional option for PPI activities alongside face-to-face and telephone meetings for all PPI contributors. Researchers and PPI contributors need to explore how best to integrate those formats and ensure parity of ability, and capacity, to contribute based on individual contributors’ needs and wants. However, our work highlights, that in- person contact remains a necessary strategy at the start of the PPI journey to build the necessary trust and rapport. The key lesson is that as researchers, we need to be as flexible and accommodating as possible to the specific and changing needs of the contributors within individual context. Inclusivity and its facilitation, particularly when dealing with the most vulnerable people in society is at the heart of involvement.

## Data Availability

Not applicable.

## Abbreviations

NIHR: National Institute of Health Research
GMPSTRC: Greater Manchester Patient Safety Translational research Centre
PPI: Patient and public involvement
